# Generation of a genomic tiling array of the human Major Histocompatibility Complex (MHC) and its application for DNA methylation analysis

**DOI:** 10.1186/1755-8794-1-19

**Published:** 2008-05-30

**Authors:** Eleni M Tomazou, Vardhman K Rakyan, Gregory Lefebvre, Robert Andrews, Peter Ellis, David K Jackson, Cordelia Langford, Matthew D Francis, Liselotte Bäckdahl, Marcos Miretti, Penny Coggill, Diego Ottaviani, Denise Sheer, Adele Murrell, Stephan Beck

**Affiliations:** 1 The Wellcome Trust Sanger Institute, Genome Campus, Hinxton, Cambridge, CB10 1SA, UK; 2 Institute of Cell and Molecular Science, Barts and The London School of Medicine and Dentistry, 4 Newark Street, London, E1 2AT, UK; 3 Cancer Research UK Cambridge Research Institute, Li Ka Shing Centre, Robinson Way, Cambridge, CB2 0RE, UK; 4 UCL Cancer Institute, University College London, 72 Huntley Street, London, WC1E 6BT, UK; 5 Cancer Research UK London Research Institute, Lincoln's Inn Fields, London, WC2A 3PX, UK

## Abstract

**Background:**

The major histocompatibility complex (MHC) is essential for human immunity and is highly associated with common diseases, including cancer. While the genetics of the MHC has been studied intensively for many decades, very little is known about the epigenetics of this most polymorphic and disease-associated region of the genome.

**Methods:**

To facilitate comprehensive epigenetic analyses of this region, we have generated a genomic tiling array of 2 Kb resolution covering the entire 4 Mb MHC region. The array has been designed to be compatible with chromatin immunoprecipitation (ChIP), methylated DNA immunoprecipitation (MeDIP), array comparative genomic hybridization (aCGH) and expression profiling, including of non-coding RNAs. The array comprises 7832 features, consisting of two replicates of both forward and reverse strands of MHC amplicons and appropriate controls.

**Results:**

Using MeDIP, we demonstrate the application of the MHC array for DNA methylation profiling and the identification of tissue-specific differentially methylated regions (tDMRs). Based on the analysis of two tissues and two cell types, we identified 90 tDMRs within the MHC and describe their characterisation.

**Conclusion:**

A tiling array covering the MHC region was developed and validated. Its successful application for DNA methylation profiling indicates that this array represents a useful tool for molecular analyses of the MHC in the context of medical genomics.

## Background

The major histocompatibility complex (MHC) is a 4 Mb region on the short arm of human chromosome 6 [1]. It is one of the most gene-dense regions of the human genome and it is associated with many complex diseases including infectious, autoimmune and inflammatory diseases as well as cancer. In many cases, their aetiologies are polygenic and involve genetic, epigenetic and environmental factors. Although past studies have generated extensive data for the genetics of the MHC resulting in important contributions to medicine [2-4] further studies are necessary to improve our understanding of the causes of such diseases. Because of its central role in so many complex diseases, elucidating the epigenetic code of the MHC can be expected to be highly beneficial for biomedical research.

Epigenetics is a term used to describe mitotically and, in some cases, meiotically heritable states of gene expression that are not due to changes in the DNA sequence [5]. The best-studied epigenetic marks are DNA methylation and histone modifications. The latter are post-translational modifications and occur at specific positions within the amino-terminus of histone tails. They include acetylation, methylation, phosphorylation, ubiquitination and other modifications and are correlated with chromatin accessibility and transcriptional activity or repression [6,7]. DNA methylation on the other hand involves the addition (or removal) of methyl groups at the carbon-5-position of cytosine. In mammals this occurs predominantly in the context of cytidine-guanosine (CpG) dinucleotides [8], but non-CpG methylation has also been reported in certain cell types and is common in plants [9-11]. In mammalian somatic cells, about 70% of CpGs are methylated (hypermethylated) and these sites predominantly occur in repetitive DNA elements, satellite DNAs, non-repetitive intergenic DNA and exons [8]. In contrast, the CpGs located in the estimated 29,000 CpG islands, found spanning the promoters and 5'-untranslated regions (5'-UTRs) of about 60% of human genes are largely unmethylated (hypomethylated) [8]. DNA methylation can regulate transcription either directly by interfering with transcription factor binding or indirectly via methyl binding domain (MBD) containing proteins resulting in changes in chromatin architecture [12,13]. Recently, non-coding RNAs (ncRNAs) have been recognised as an additional component associated with epigenetic modulation and have been reported to be involved in X-chromosome inactivation, chromatin structure, DNA imprinting and DNA demethylation [14].

Emerging evidence suggests that epigenetic events are associated with the regulation of MHC gene expression. It has been shown, for instance, that the MHC class II transactivator (CIITA) and the regulatory factor X (RFX) proteins serve as focal points for recruiting histone modifying enzymes to MHC class II promoters, whereby CIITA itself is regulated by DNA methylation, histone modifications and ncRNAs [15,16]. Treatment of melanoma and esophageal cell lines with the DNA methylation inhibitor 5-aza-2-deoxycytidine led to restoration of MHC class I expression (which is suppressed in these cell lines), implicating DNA methylation in expression of MHC class I genes [17-19].

As part of the Human Epigenome Project (HEP), about 2.5% of the MHC region has been analysed for DNA methylation [20]. This study has demonstrated that a significant proportion (10%) of the MHC loci analysed show tissue-specific DNA methylation profiles. Such regions have been termed tissue-specific differentially methylated regions (tDMRs) and are thought to contain elements involved in tissue-specific gene expression [21].

To facilitate a more comprehensive epigenetic analysis of the MHC, we have constructed a tiling array that covers the entire 4 Mb of the MHC at 2 kb resolution. This array is an economical alternative to commercial arrays and can be used for: i) ChIP-on-chip studies, investigating DNA/protein interactions [22]; ii) DNA methylation studies, investigating tissue- or disease-specific DNA methylation profiles [23,24]; iii) array comparative genomic hybridization (aCGH), investigating copy number variations (CNVs) [25,26]; and finally, for expression studies, investigating both coding and non-coding RNAs.

Here we describe the generation and properties of an array for the human MHC and we show how it can be used for DNA methylation studies, particularly for the identification of DMRs.

## Methods

### Design, generation and quality control of the MHC tiling array

The array was designed to cover the entire MHC region as a minimally overlapping tile path, with appropriate controls. A total of 1747 overlapping plasmid clones were used to generate the array. Of those, 1662 clones (average insert size 2 kb) were picked from the HapMap chromosome 6 library [27] and 85 clones were generated by cloning gap-spanning PCR amplicons (average insert size 332 bp). Some repeat-rich regions (about 12 kb in total) proved to be refractory to PCR amplification and are hence missing from the array. Therefore, the total coverage represents 99.67% of the MHC region. In addition, we generated and included 43 PCR-derived clones as controls, covering: i) CpG islands of BRCA1, GSTP1, RARB2 and MLH1 genes [28]. ii); imprinted regions (H19, IGF2, KvDMR1, HSIGF2G, IGF2RDMR2 and DMR0) [29]; iii) gene poor regions of chromosome 6; iv) matrix attachment regions of the *β-globin *gene cluster [30]; v) loop-associated DNA of the PRM2 gene [30]; vi) promoter regions of the GAPDH and IRF1 genes; vii) replication origin of the LB2 gene; vii) replication origin-lacking region of the *β-globin *locus; and viii) DNAase I-hypersensitivity sites of the *β-globin *locus control region. Ten genes from the Arabidopsis genome (spotted in replicates, distributed across the array) that can be used to assign DNA barcodes as internal controls were also included. In addition, 192 Cy3 spots were printed on each array that can be used for calibration and orientation. Except for the Cy3 spots, none of other controls were used for the analysis described here but may be useful for other types of analyses. MHC probe coordinates and primer sets used for the generation of gap-spanning and control clones can be provided upon request.

Double-stranded amino-linked amplicons were generated from each clone using vector-specific PCR in 50 mM KCl, 5 mM Tris pH 8.5 and 2.5 mM MgCl_2 _(10 min at 95°C; followed by 35 cycles of 95°C for 1 min, 60°C for 1.5 min, 72°C for 7 min; and a final extension of 72°C for 10 min – Forward primer 5'-CCCAGTCACGACGTTGTAAAACG-3', Reverse primer 5'-AGCGGATAACAATTTCACACAGG-3'). In order to generate strand-specific array probes, two separate PCR reactions were performed for each clone, in one case using a 5'-aminolinked primer for the forward strand, and in the other case, for the reverse strand. After quality assessment of the products by gel electrophoresis, spotting buffer was added directly to a final concentration of 250 mM sodium phosphate pH 8.5, 0.00025% w/v sarkosyl, 0.1% sodium azide, and the products were filtered (Multiscreen-GV filter plates, Millipore). Arrays were spotted onto amine binding slides (CodeLink, GE Healthcare) at 20–25°C, 40–50% relative humidity. After an overnight incubation in a humid chamber, the slides were blocked (1% ammonium hydroxide for 5 min, followed by 0.1% SDS for 5 min) and denatured (95°C ddH_2_O for 2 min), rinsed in ddH_2_O and dried by centrifugation for 5 min at 250 × g. Thus, the covalently attached strand-specific probes were rendered single-stranded in preparation for hybridization.

The final array therefore comprises 7832 features (2 × 1747 MHC forward probes, 2 × 1747 MHC reverse probes, 4 × 43 human control probes, 480 Arabidopsis control probes and 192 Cy3 dye controls). Resequencing of 240 probes (15% of total) identified 7 probes that failed to match to the expected reference sequences. Aliquots of all probes can be made available upon request for further QC analysis. From this partial analysis, we extrapolate that about 97% of the probes are correct and should be informative.

### DNA samples

Human DNA samples from healthy individuals were obtained from AMS Biotechnology (Oxon, UK), Analytical Biological Services (Wilmington DE, USA) and from the MHC Haplotype Project [31]. Samples included DNA extracted from two tissues (liver and placenta) and 2 cell types (CD8^+ ^lymphocytes and sperm). Additional information on those samples is summarized in Table 1.

**Table 1:** Tissues and cell types used in this study

**Index**	**Tissue**	**Replicate**	**Age (yrs)**	**Sex**	**Ethnicity**	**Supplier**
1	Liver	1	37	M	Caucasian	ABS, Wilmington, DE, USA
2	Liver	2	29	M	Caucasian	BCI, Haywatd, CA, USA
3	Placenta	1	29 (mother)	F	Caucasian	ABS, Wilmington, DE, USA
4	Placenta	2	31 (mother)	F	Caucasian	ABS, Wilmington, DE, USA
5	Sperm	1	20–49	M	Caucasian	MHC Haplotype Project [31]
6	Sperm	2	20–49	M	Caucasian	MHC Haplotype Project [31]
7	T-cells CD8	1	41	M	Caucasian	ABS, Wilmington, DE, USA
8	T-cells CD8	2	27	F	African American	ABS, Wilmington, DE, USA

ABS: Analytical Biological Services [50]

BCI: BioChain Institute [51]

### Methylated DNA immunoprecipitation (MeDIP)

MeDIP was performed as described by Weber and colleagues [23] with the following modifications. DNA samples (2.5 μg) were sheared into fragments of average size of 600 bp using a sonicator (Virtis). Fragmented DNA was incubated with 1 × buffer 2 (New England Biolabs, UK), 10 × BSA (NEB, U.K.), 1.2 μl dNTP mix (10 mM each) (Abgene, UK), 3 Units of T4 DNA polymerase (New England Biolabs, U.K.) and distilled water to a final volume of 120 μl for 20 minutes at 12°C. The reaction was cleaned up using a Zymo-5 kit (Genetix, U.K.) according to the manufacturer's instructions but the final elution was done in 30 μl of TE buffer (10 mM Tris-HCl pH 8.5, 1 mM EDTA). The adaptors JW102 (5'-gcggtgacccgggagatctgaattc-3') and JW103 (5'-gaattcagatc-3') were ligated to the cleaned-up DNA by incubation overnight at 16°C in a reaction containing 40 μl adaptor mix (50 μM), 6 μl T4 DNA ligase 10 × buffer (NEB, UK), 5 μl T4 DNA ligase (400 U/μl) (NEB, U.K.) and distilled water to a final volume of 100 μl. DNA was cleaned up as described above. To fill in the overhangs, the sample DNA was incubated at 72°C for 10 minutes with 1 μl dNTP mix (10 mM each), 5 μl 10 × AmpliTaq Gold PCR buffer (Applied Biosystems – Roche), 3 μl MgCl_2 _(250 mM), 5 U AmpliTaq Polymerase and distilled water to a final volume of 50 μl. DNA was cleaned up as described above. 50 ng of the ligated DNA sample was set aside as the input fraction. 1.2 μg of the ligated DNA sample was denatured for 10 minutes at 100°C and then placed on ice for 5 minutes. Immunoprecipitation was performed in 1 × IP buffer (20 mM sodium phosphate pH 7, 280 mM NaCl, 0.1% Triton X-100) and 3 μl of 5-MeC-mAb (Eurogentec) with incubation at 4°C with slow rotation for 2 hours. 10 μl Dynabeads (M-280 Sheep anti-Mouse IgG – 6.7 × 10^8 ^beads/ml) (Dynal Biotech) were washed in 1 × IP buffer according to the manufacturer's instructions and added to the DNA-antibody mixture and then incubated at 4°C with slow rotation for 2 hours. The Dynabead-Ab-DNA mixture was washed three times with 500 μl IP buffer and finally resuspended in 100 μl of proteinase K buffer (10 mM Tris-HCl pH 7.8, 5 mM EDTA, 0.5% SDS). 1 μl of proteinase K (50 U/ml) (Roche Diagnostics) was added and incubated at 50°C for 2 hours with rotation. The sample was cleaned up using a Zymo kit-5 (using 700 μl binding buffer). The DNA concentration was determined with a NanoDrop (using 1 OD_260 _= 33 μg) and diluted to 1 ng/μl. Two separate amplifications were performed for the respective IP and input fractions using ligation-mediated PCR (LM-PCR) [32]. LM-PCR was performed in a final volume of 50 μl containing 10 μl distilled water, 10 μl Advantage-GC buffer (BD Biosciences), 10 μl GC-melt (BD Biosciences), 3.1 μl 25 mM Mg(OAc)_2_, 5 μl JW-102 primer (10 μM), 1.4 μl dNTPs (10 mM each), 1 μl Advantage-GC polymerase (BD Biosciences) and 10 μl DNA (1 ng/μl). Reaction conditions were as follows: 1 cycle at 95°C for 2 minutes for initial denaturation, 20 cycles at 94°C for 30 seconds, 68°C for 3 minutes and 1 cycle at 68°C for 10 minutes. After LM-PCR, the reactions were cleaned up using a QIAquick PCR Purification kit (Qiagen) and eluted with 50 μl of water (pre-heated to 50°C).

### Real-time PCR of MeDIP samples

For MeDIP validation, we performed quantitative real-time PCR (qRT-PCR), using an ABI Prism 7300 Sequence Detection System and 30 ng of input and immunoprecipitated DNA (after LM-PCR). For each qRT-PCR reaction (total volume of 13.5 μl) we used 6.5 μl SYBR Green PCR master mix (Eurogentec) and 2.5 μl primer mix (1.5 μM each.). Reaction conditions were as follows: 1 cycle at 50°C for 2 minutes, 1 cycle at 95°C for 10 minutes, 40 cycles at 95°C for 15 seconds and 1 cycle at 60°C for 1 minute. Reactions were done in triplicates. To evaluate the relative enrichment of target sequences after MeDIP, we normalized (for each amplicon tested) the C_t _of the MeDIP fraction to the C_t _of the input (ΔC_t_). Subsequently we normalised the ΔC_t _of each target sequence to the ΔC_t _of an unmethylated control sequence (ΔΔC_t_). Finally, we calculated the enrichment $\left(\text{E}=2^{\Delta \Delta {\text{C}}_{\text{t}}}\right)$. Primer sequences can be provided upon request.

### DNA labelling and microarray hybridization

Fluorescent labelling was performed using a modified Bioprime labelling kit (Invitrogen) in a 130.5 μl reaction containing 100 ng DNA, 15 μl dNTP mix (2 mM dATP, 2 mM dTTP, 2 mM dGTP, and 0.5 mM dCTP), and 1.5 μl Cy5/Cy3 dCTP (1 mM) (Perkin Elmer). The reactions were purified using Micro-spin G50 columns (Pharmacia-Amersham) in accordance with the manufacturer's instructions. Reference and test samples were combined and precipitated with 55 μl of 3 M sodium acetate (pH 5.2) in 2.5 volumes of ethanol with 135 μg human C_o_t1 DNA (Invitrogen). The DNA pellet was resuspended in hybridization buffer containing 50% deionized formamide, 10% dextran sulphate, 10 mM Tris-HCl (pH 7.4), 2 × SSC, 0.1% Tween-20, and 200 μg yeast tRNA (Invitrogen). Hybridization was performed for 24 hours at 37°C on a MAUI hybridization platform. Finally, the arrays were washed serially in solution 1 (2 × SSC, 0.03% SDS) for 5 minutes at room temperature, in solution 1 for 5 minutes at 60°C, four times in solution 2 (2 × SSC) for 20 minutes at room temperature, in solution 3 (PBS, 0.05% Tween20) for 10 minutes at room temperature and finally in HPLC water for 10 minutes at room temperature. Subsequently the arrays were dried and scanned using a ScanArray Express HT scanner (PerkinElmer).

### Microarray data analysis

For each sample we analysed two biological replicates. All hybridizations were performed with fluorochrome-reversed pairs of two-colour labelled probes (dye swaps). For the purpose of this analysis we treated the forward and reverse probes as replicates. Hence, for each sample tested, we obtained 16 measurements derived from quadruplicate spots on 4 array hybridizations (two biological replicates plus dye swaps). Fluorescence intensities were determined using the ScanArray Express software (PerkinElemer). Fusion of dye-swap and biological replicate results and subsequent analyses were performed using Bioconductor [33]. For each probe, log-ratios were normalised within arrays using a Local Linear Regression (loess) [34] whereas average intensities were normalised between arrays [35] leaving previously normalised ratios unchanged. Dye-swapped samples and biological replicates were defined in a design matrix. Subsequent analyses were performed according to the design matrix by fitting a linear model to log-ratios. The fit is by generalized least squares, allowing for correlation between the four duplicated spots [36]. Finally ranking the features according to their evidence of discrepancy between effects defined in the design matrix has been performed by using empirical Bayes method [37]. The array data described here have been deposited in ArrayExpress under accession numbers E-TABM-471 (experiment) and A-MEXP-1163 (array design).

### tDMR feature analysis

The Application Programme Interfaces (API) was used to extract genomic features associated with tDMR coordinates from the Ensembl functional genomics dataset (NCBI36). The whole of chromosome 6 was scanned using a 2 kb window and 1 kb steps (i.e. moving the window from the start to the end of the chromosome, shifting each time by 1 kb). For each window, the number of each type of feature within the bounds of the window was counted. This way, a discrete probability distribution was generated, which determines, for a randomly selected window, how likely it would be to have a certain number of features. Windows that overlapped a gap in the assembly were ignored to avoid biasing the result. For each DMR and for each type of feature, the number of features that were found and their probability distribution were used to calculate (using 95% confidence interval) if the DMR was enriched for that feature.

### Bisulphite sequencing

Genomic DNA was subjected to sodium bisulphite conversion using the EZ DNA methylation Kit (Genetix, U.K.) according to the manufacturer's instructions. Primer design, bisulphite-PCR and sequencing were carried out as described by Rakyan et al., 2004 [20]. Primer sequences can be provided upon request. Absolute DNA methylation values were estimated from signal ratios of the corresponding sequence traces using the ESME software [38].

## Results

### MHC tiling array

In order to facilitate analyses of the regulation and function of genes and control elements within the MHC region on chromosome 6, we constructed a tiling array that encompasses the almost (99.67%) complete 4 Mb region at 2 kb resolution. As described in the Methods section, the array entails a total of 7832 features (7640 probes and 192 Cy3 control spots) of which 97% are estimated to be informative following the quality control described under Methods. The array can be requested from the Microarray Facility at the Wellcome Trust Sanger Institute [39].

### Generation of DNA methylation profiles

**Figure 1:**
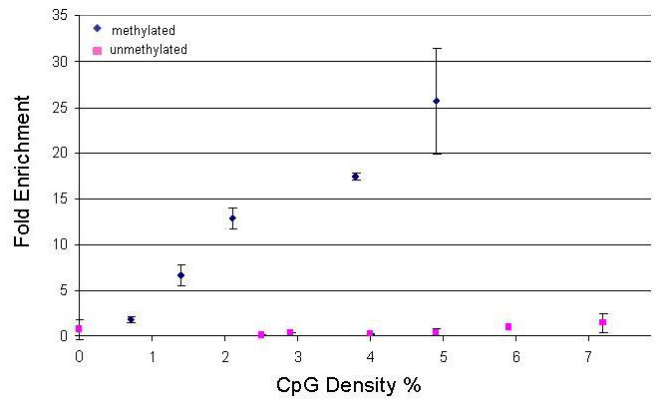
**Correlation between enrichment after MeDIP and CpG density**. Control sequences that are methylated, unmethylated or lack CpG sites were selected from HEP [49]. MeDIP was done using liver genomic DNA. The relative enrichment of the MeDIP versus input fractions was calculated based on qRT-PCR data. The graph shows a specific and efficient enrichment of methylated over unmethylated fractions. The error bars indicate the variance of two independent measurements. Methylated amplicons display an approximately linear dependency on CpG density (CpG density equals the number CpG sites per amplicon divided by the length of the amplicon multiplied by 100).

To demonstrate the utility of the MHC tiling array, we first generated comprehensive DNA methylation profiles in conjunction with the Methylated DNA Immunoprecipitation (MeDIP) assay [23]. Using an antibody that specifically recognises 5-methylcytosine, we immunoprecipitated the methylated fraction of sheared genomic DNA from two tissues (liver and placenta) and two cell types (CD8^+ ^lymphocytes and sperm). MeDIP and input fractions were amplified by ligation-mediated PCR (LM-PCR) [32]. We validated MeDIP by performing qRT-PCR (see Methods) to test the enrichment of regions with varying CpG densities for which the methylation status was known from the Human Epigenome Project [20,40]. Figure 1 shows that following MeDIP, methylated regions are enriched approximately proportionally to their CpG densities and no significant enrichment irrespective of CpG density is observed for unmethylated regions. Using a threshold of ≥5-fold enrichment, the MeDIP assay is therefore sensitive for regions of ≥1% CpG density.

**Figure 2:**
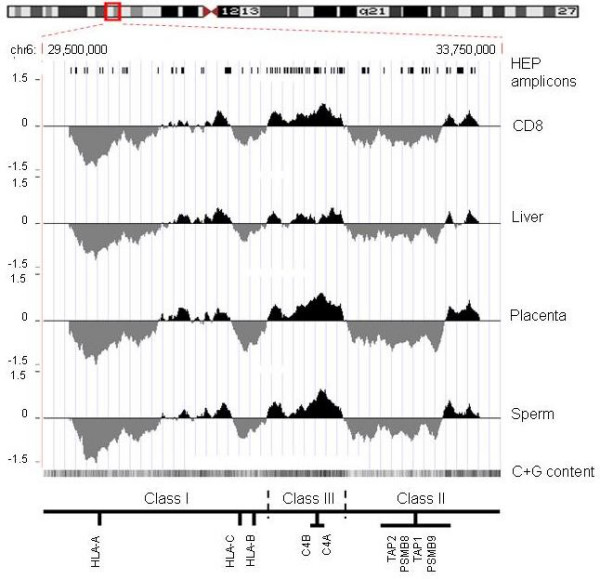
**DNA methylation profiles of the MHC**. For each of the four samples tested (CD8^+ ^lymphocytes, liver, placenta, sperm), the log_2 _signal ratios (MeDIP/input) were uploaded as individual tracks to the UCSC genome browser using the 'smooth' function. Regions enriched or depleted in DNA methylation are shaded in black and grey, respectively. Also shown are the locations of HEP amplicons [49] and a track of the C+G content (the darker the shading, the higher the C+G content). For orientation, the approximate positions of the MHC class I, II and II sub-regions and some landmark genes are indicated.

Using this threshold (actual enrichment range was 5–80 fold), we generated DNA methylation profiles of the entire MHC for CD8^+ ^lymphocytes, sperm, liver and placenta (Figure 2). Control hybridizations assessing biological replicates (R^2 ^> 0.97), dye-swaps (R^2 ^> 0.72) and LM-PCR (R^2 ^> 0.88) showed that any bias introduced by these factors was within an acceptable range (Additional File 1). At this (megabase) resolution, three main observations can be made: (i) The overall profiles correlate significantly (0.83 < R^2 ^< 0.93), suggesting few or no large-scale (>100 Kb) differences in DNA methylation, except perhaps in liver, where some regions appear to be lower in methylation than in other tissues. (ii) As expected from the result shown in Figure 1 (although CpG density was analysed here), the profiles correlate very well with C+G content, clearly demarcating the boundaries of the MHC class I, III, II and extended class II regions. (iii) The profiles further show the vast improvement in coverage compared to the 253 amplicons, analysed as part of the Human Epigenome Project [20].

**Figure 3:**
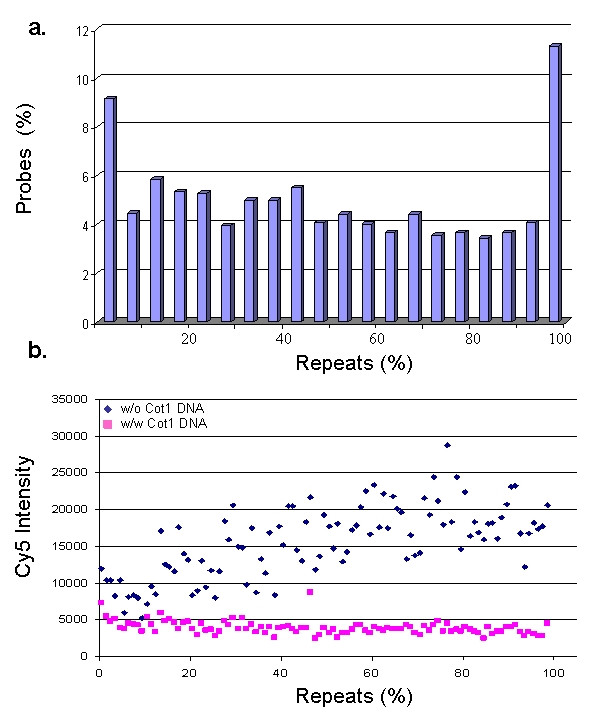
**Distribution and suppression of repeat sequences**. a) Distribution (in 5% bins) and frequency of repeat sequences within probes on the array. b). Suppression of repeat-specific signal using Cot1 DNA. Two independent hybridizations were carried out using genomic DNA extracted from CD8^+ ^lymphocytes. In both experiments total DNA was labelled with Cy5 dye. Only in one of them unlabelled Cot1 DNA was added. In the non-Cot1 hybridization, Cy5 intensity increases almost linearly with repeat density until it reaches a plateau (around 25,000 Cy5 intensity). In the presence of Cot1 DNA, Cy5 intensity of highly repetitive probes is comparable to those of repeat-free probes. Repeats were defined based on the 'All repeats' track in Ensembl browser [43].

Compared to most commercial and custom arrays, our tiling array also contains repeat elements, allowing such sequences to be analysed as well if desired. Figure 3a shows the distribution and frequency of repeat sequences within the probes on the array. About 9% of the probes have low (0–5%) repeat content and around 11% have high (95–100%) repeat content. The majority (80%) of probes have a random repeat content ranging from 6–94%. For studies that are not designed to interrogate repeat sequences (as the study presented here) we show that repeat sequences can be efficiently blocked by the addition of human Cot1 DNA during hybridization (Figure 3b). For that, we compared the probe intensities of the Cy5 channel for two hybridizations, one with and the other without Cot1 DNA. In the presence of Cot1 DNA, the intensities of repeat-containing probes are clearly reduced to the same level detected for repeat-free probes, indicating that undesired repeat signals can be blocked and that the unique parts of repeat-containing probes remain to be informative and can be kept for further analysis.

### Identification and characterisation of tDMRs

**Figure 4:**
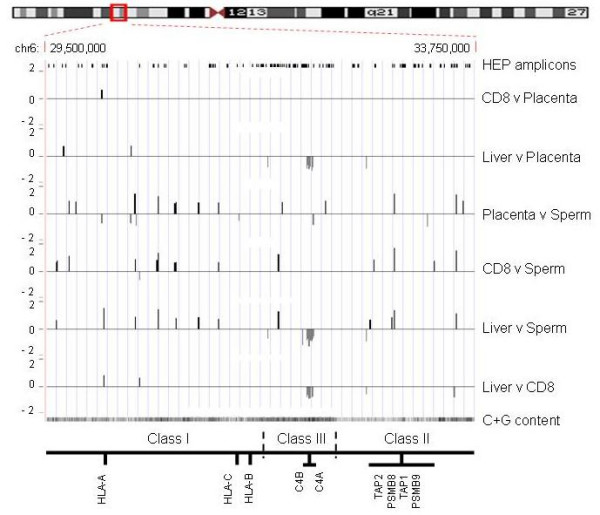
**tDMRs within the MHC region**. Pair-wise comparisons (six in total) of the MHC array-derived DNA methylation profiles were performed using t-statistics. A threshold of p-value < 0.001 was used. In total 90 tDMRs were identified. Vertical axis shows the log_2 _ratio of the two corresponding methylation profiles. Each line represents a tDMR (average size 2 kb). Black lines represent tDMRs that are more methylated in sample 1 of the comparison and grey boxes represent tDMRs that are more methylated in sample 2 of the comparison (the identities of the pair-wise comparisons are given on the right). The majority of tDMRs are present in comparisons with sperm. The locations of HEP amplicons, a track of the C+G content and the approximate positions of the MHC class I, II and II subregions and some landmark genes are also indicated. Class III region encoding for the C4 genes seems to be less methylated in liver.

**Figure 5:**
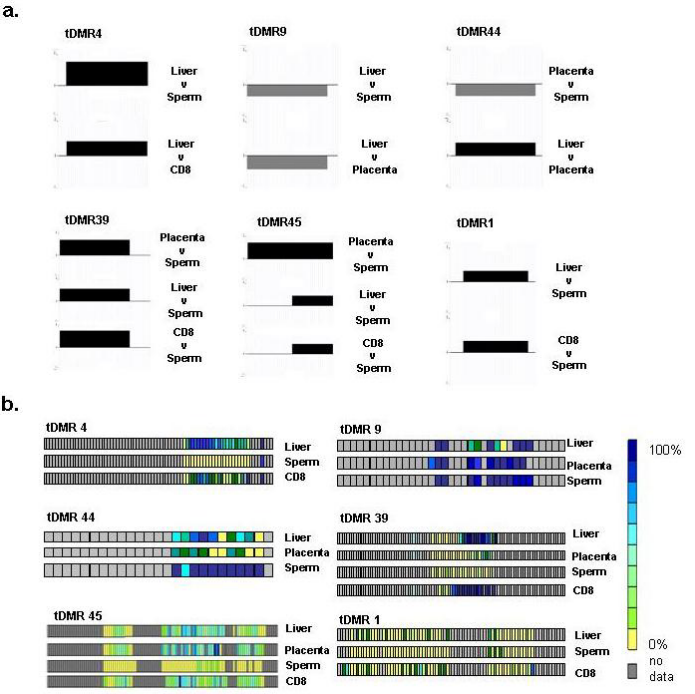
**tDMR validation**. Six tDMRs were randomly selected and subjected to bisulphite sequencing analysis. a). tDMR status based on pair-wise comparisons of the log_2 _MeDIP enrichment ratios of the indicated tissues/cell types. Black boxes represent tDMRs that are more methylated in sample 1 of the comparison and grey boxes represent tDMRs that are more methylated in sample 2 of the comparison. b). Absolute DNA methylation values of individual CpG sites in tDMRs based on bisulphite sequencing analysis. Because of assay and or technical limitations, bisulphite data could only be obtained for about 50% of the CpG sites involved in the putative tDMRs. Each square represents a CpG site. The colour code indicates methylation values as calculated by ESME (see Methods). Grey squares indicate CpG sites for which no data could be obtained. Based on this analysis, bisulphite data essentially agree with array data in all cases. Numbers of tDMRs correspond to tDMR numbers in table 2.

For the identification of tDMRs, we performed pair-wise comparisons (six in total: CD8^+ ^lymphocytes versus placenta, liver versus placenta, placenta versus sperm, CD8^+ ^lymphocytes versus sperm, liver versus sperm, and liver versus CD8^+ ^lymphocytes) of the array-derived DNA methylation profiles. At 2 kb, the probe resolution was not high enough to determine if more than one tDMR was contained within a probe or if positive, adjoining probes were part of the same tDMR. Therefore, each differentially methylated probe was considered to be a separate tDMR. According to this definition, we identified a total of 90 tDMRs of which 35 were present in more than one comparison (Figure 4; Additional File 2). For validation, we randomly selected six tDMRs (irrespective of their genomic functionality) and subjected them to independent methylation analysis using bisulphite DNA sequencing. Figure 5 shows their methylation status based on comparison of their respective MeDIP array profiles (a) and their absolute methylation values based on bisulphite sequencing (b). The characteristics of these tDMRs are shown in Table 2. In all six cases, the bisulphite sequencing results were consistent with the array data, indicating that that the array is suitable for the identification of tDMRs.

**Table 2:** Genomic features of non-redundant tDMRs

	**chr6 coordinates (NCBI_35)**	**TSS**	**CTCF**	**H4K20 me1**	**PolII**	**H3K4 me2**	**H3K36 me3**	**H3K4 me3**	**DnaseI**	**H3K4 me1**	**CpG island**	**ECR**	**H2AZ**	**repeats %**	**CpG %**
1	29823989–29826356		x					x	x		x		x	28.63	2.94
2	30000805–30003606				x			x	x					12.6	9.28
*3	30228982–30231712													26.44	2.64
*4	30247370–30249040		x					x	x		x			4.73	9.1
*5	30565890–30568365			x	x		x	x						11.95	4.77
6	30721858–30724158							x					x	5.91	10.52
7	30731648–30734384													42.71	2.27
8	30891136–30893651													95.08	5.24
9	31709197–31711626											x		79.93	5.01
*10	31803609–31806450		x					x	x			x		0	2.88
11	31841070–31843352											x		0	11.05
12	32020686–32023216											x		18.4	5.61
13	32056738–32058031	x												6.57	3.935
*14	32067481–32068550													0	3.09
*15	32071709–32072864													0	5.61
16	32073608–32074514													0	5.02
17	32074474–32074660													13.67	3.97
*18	32077678–32079121													0	5.35
*19	32081199–32081780													19.53	5.4
20	32088659–32090434													0	3.685
*21	32088718–32090526													0	3.41
*22	32090749–32092076													1.96	4.22
23	32092057–32093147													0	2.38
24	32094350–32095101													100	1.33
25	32098656–32099323													100	3.59
26	32099573–32100214													0	3.875
*27	32104734–32105602													0	5.29
*28	32107212–32107398													0	5.35
29	32109195–32110435													9.995	5.6
*30	32110416–32111859													19.53	5.4
31	32115381–32116535											x		0	7.27
32	32119000–32120024											x		0	7.61
33	32223988–32226638							x			x			4.19	11.09
34	32659407–32660508							x	x		x			9	5
35	32817677–32820582													20.44	2.96
36	32836042–32838492													9.42	7.26
37	33192620–33193912		x											32.79	6.5
*38	33372651–33375048			x	x	x	x	x	x	x		x		10.93	8.76
39	33389687–33392295				x			x				x		2.3	7.51
40	29830203–29832660													6.08	7.77
41	29889483–29892066													21.2	4.15
42	29937894–29939594													38.78	2.48
43	30484481–30486798													96.59	1.04
44	30491424–30493923													1.4	2
45	30526624–30528439							x	x					3.54	7.33
46	30527803–30529467		x					x						3.54	6.73
47	30534798–30537070													42.42	1.26
48	30881555–30884300													98.55	4.27
49	31092038–31094660													74.72	7.51
50	31270669–31273172		x					x						4.59	2.75
51	31454436–31456982													8.59	5.84
*52	32590480–32591619													44.47	2.11
53	32622631–32625110													91.09	5.97
54	33132309–33134479								x					24.37	1.01
55	33450625–33452501													84.66	2.88

A total number of 55 non-redundant tDMRs were identified. tDMR co-ordinates on chromosome 6 are provided. tDMRs 1–39 are intragenic and 40–55 intergenic. Enrichment of genomic features, including CpG islands, DNAseI sites, TSSs, ECRs, CTCF binding sites, RNA PolII binding sites, histone marks (H4K20me1, H3K4me2, H3K4me3, H3K36me3, H3K4me1) and H2AZ was tested and marked by symbol 'x' if enrichment was statistically significant (P < 0.05). Percent CpG and repeat density were also determined and are shown for each tDMR. tDMRs 14 – 30 (intragenic) and 51 (intergenic) are mapping to the region encoding for C4A and C4B genes. Asterisks indicate the tDMRs that overlap with Affy_U95 expression array probes.

According to the pair-wise analyses, sperm is most frequently differentially methylated which agrees with the findings of the Human Epigenome Project [40]. The majority of tDMRs identified in sperm are hypomethylated compared to the other samples (65% of tDMRs in placenta-sperm comparison; 93% of tDMRs in CD8-sperm comparison; 32% of tDMRs in liver-sperm comparison). Notable exceptions are the tDMRs identified in the complement region which seem to be less methylated in liver than any of the other samples (Figure 4; Additional File 2).

**Figure 6:**
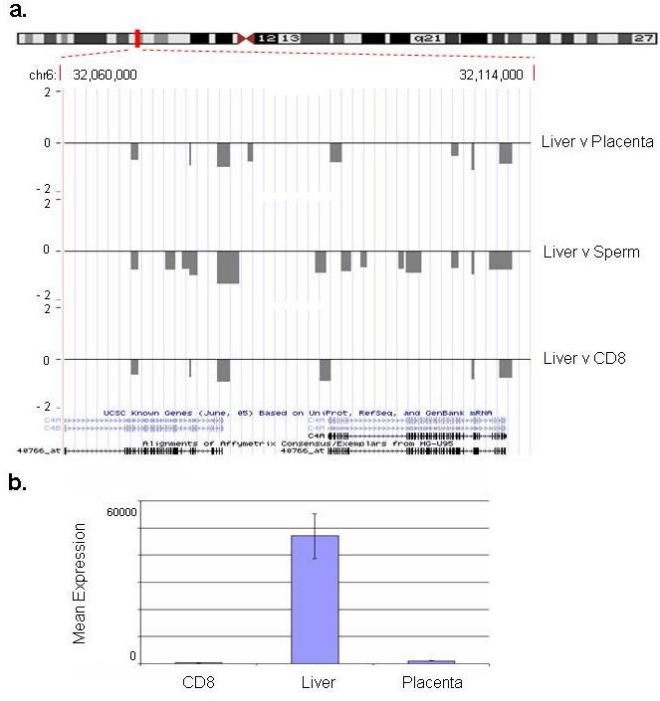
**Example of tDMRs correlating with tissue-specific gene expression**. a) tDMRs within the region encoding the C4A and C4B genes. Vertical axis shows the log_2 _ratio of the two corresponding methylation profiles. Grey lines indicate regions (average size 2 kb) that are less methylated in liver compared to the other samples (placenta, sperm, CD8). Known genes and Affy_U95 expression array probes within this region are also shown. b). Expression of C4A and C4B. Graph shows the mean expression values of the probe corresponding to C4A and C4B (Affy_ID: 40776_at) transcripts in three samples tested: CD8, liver, placenta. C4A and C4B transcripts are highly expressed in liver tissue only. Data were taken from the GNF SymAtlas [41].

Next, we correlated the tDMRs with gene expression using data publicly available from the Genomics Institute of the Novartis Research Foundation Gene Expression Atlas database [41]. This database contains whole-genome mRNA expression data obtained using human U95A Affymetrix microarray chips [42] and mRNA extracted from a number of tissues, including liver, placenta and CD8^+ ^lymphocytes (sperm was not included in this database). We identified 7 probes on the U95A Affymetrix array that overlap with tDMRs identified in our liver versus placenta, liver versus CD8^+ ^lymphocytes and CD8^+^lymphocytes versus placenta comparisons. Genomic features of these tDMRs are shown in Table 2 (see below). One of the probes (Affymetrix ID 40766_at that corresponds to C4A and C4B transcripts) shows a high inverse correlation between expression and methylation at these loci (Figure 6). Both loci are highly expressed and hypomethylated in liver.

**Figure 7:**
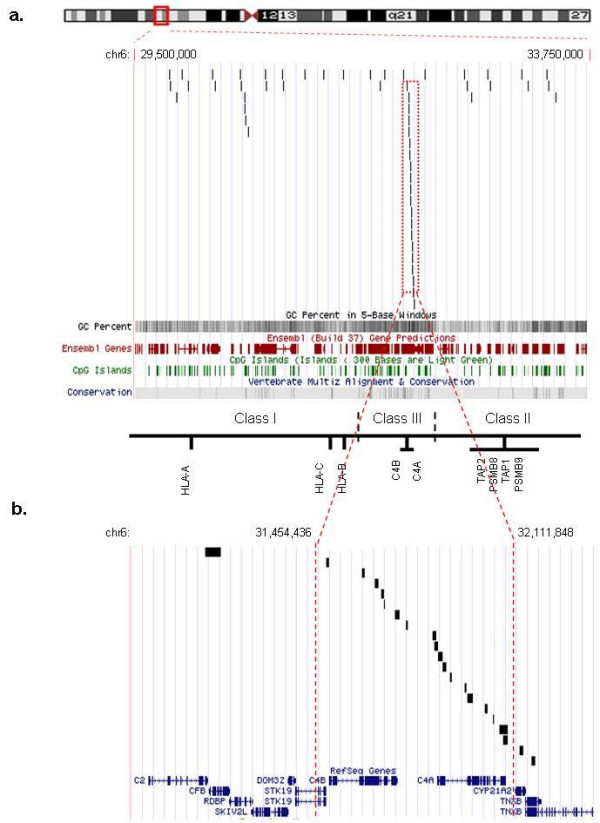
**Non-redundant tDMRs within the MHC region**. a). Screen-shot showing the locations of 55 non-redundant tDMRs identified in the MHC region after uploading of the data to the UCSC genome browser. Each vertical black line represents a putative tDMR. The high density of 18 tDMRs within the C4A and C4B complement region is clearly visible (boxed with red doted line). Tracks showing C+G content, Ensembl genes, CpG islands and conservation are also shown. b). Enlargement of the C4A and C4B complement region showing the 18 overlapping or adjacent tDMRs (delimited by red dotted lines) which could be part of one large tDMR spanning the entire C4 complement region.

35 out of the 90 identified tDMRs were observed in more than one comparison. Hence there are 55 loci (average size 2 kb) within the MHC region that according to this study shows tissue-specific methylation levels. We define these 55 loci as non-redundant tDMRs (to reflect the non-redundancy at the sequence level) and show their genomic locations in Figure 7 and Table 2. The high density of 18 non-redundant tDMRs within the C4 complement region is clearly visible. To characterize their potential functionality, the 55 non-redundant tDMRs were analyzed for a number of genomic features using the ENSEMBL functional build [43]. The result of this analysis is summarized in Table 2. We found the majority (39) of these tDMRs to map to intragenic regions and the minority (16) to map to intergenic regions. While repetitive elements were overrepresented within the intergenic tDMRs (44%), DNAse I sites and evolutionary conserved elements (ECRs) were overrepresented within the intragenic tDMRs (15%). Furthermore, only 2% of the tDMRs contained transcription start sites (TSS) and about 7% CpG islands and RNA polymerase II binding sites. In all, 21% of the tDMRs contained features significantly (P < 0.05) associated with regulation, such as CpG islands, DNase1 and RNA polII binding sites, TSSs and ECRs. Although only few other epigenetic data are yet publicly available for the MHC, we also analyzed the tDMRs for features associated with epigenetic function. Based on this analysis, 6 (11%) tDMRs have insulator protein (CTCF) binding sites [44], 13 correlated with the transcription-activating histone marks (H3K4me2, H3K36me3, H3K4me3 and H3K4me1) and two with the transcription-silencing mark H4K20me1 [6]. Interestingly, 54% of the H3K4me3 sites overlapping with both intragenic and intergenic tDMRs appeared to be close to DNaseI sites. Finally, two tDMRs were associated with the histone variant H2AZ [45].

## Discussion

The array reported here is the first high-resolution (2 Kb) genomic tiling array of the entire MHC. Commercially available tiling arrays usually exclude repeat sequences and therefore cover only about 50% of the genomic sequence. Previous whole-genome tiling arrays [25] that included the MHC were constructed from P1 artificial chromosomes (PACs) and bacterial artificial chromosomes (BACs), resulting in a resolution of approximately 100 Kb. By utilizing a public clone resource [27], our array could be generated at a fraction of the costs associated with commercial arrays, albeit at lower resolution than is achievable with these platforms. The array is compatible with standard array processing and scanning platforms and contains 7832 features of which about 97% can be expected to be informative according to our quality control procedures. Upon request, the MHC array is freely available from the Microarray Facility at the Wellcome Trust Sanger Institute [39].

To demonstrate utility, we used the array for DNA methylation profiling of four samples used for the HEP study: two tissues (liver and placenta), CD8^+ ^lymphocytes and sperm. Comparison of these profiles allowed us to identify 55 putative, non-redundant tDMRs (90 in total). From these, we randomly selected 10% (6 tDMRs) for validation by an independent method. In all cases, tDMR status could be confirmed, indicating that the array is suitable for DNA methylation analysis. While the analysis carried out here is informative with respect to differential methylation between samples, it did not allow assigning absolute DNA methylation values to each tDMR. This is not a shortcoming of the array but a limitation of the MeDIP assay which is highly dependent on CpG density as illustrated in Figure 1. Therefore, it was not possible to compare our data directly with the HEP data which, in any case, only cover about 2.5% of the MHC. The on-going development of a novel algorithm employing a Bayesian de-convolution strategy to normalize MeDIP array data for CpG density is likely to overcome this current limitation in the near future (T. Down et al., personal communication). For the same reason as mentioned above, the limited number of samples did not allow us to analyse the data for inter-individual variation which was observed in the HEP study [20].

Finally, we correlated gene-associated tDMRs with expression data of the cognate genes available from the GNF SymAtlas. We found a strong correlation within the region encoding for instance the fourth component of the human complement (C4). C4 is an essential factor of the innate immunity and consists of two isoforms (C4A and C4B) that differ only in five nucleotides [46]. C4A and C4B are examples of copy number variants (CNVs) in the human genome. We show that regions within the 5'-UTR, 3'-UTR and the gene body of C4A and C4B are less methylated in liver than in sperm, placenta and CD8^+ ^lymphocytes. As these two genes are expressed only in liver, it is possible that DNA methylation is the underlying mechanism controlling their expression. At this point, sensitivity and specificity should also be considered. While sensitivity is not an issue in this case (the experimental design normalizes for the genotype of the sample DNA), specificity is. As neither our array nor the Affymetrix U95 array can discriminate between C4A and C4B (which are more than 99% identical), it was not possible to ascertain whether or not these two loci are differentially methylated in this case. Selective hypermethylation is a known mechanism for silencing of duplicated genes [47].

## Conclusion

We have generated and validated a genomic tiling array that can be used to analyse genetic and epigenetic features of the MHC. We demonstrated its utility for DNA methylation profiling and the identification of tDMRs. Based on our experience, we expect the array to be suitable for a number of assays (e.g. aCGH, ChIP-chip and expression analysis) relevant to medical genomics and are currently in the process of applying it to investigate the down-regulation of HLA class I molecules, a phenotype commonly associated with cancer [48].

## Competing interests

The authors declare that they have no competing interests.

## Authors' contributions

EMT, AM and SB conceived the study. EMT carried out the experiments. VKR, CL, LB, MM, PCC, DO and DS contributed materials. EMT, VKR, GL, RA, PE, DKJ, MDF and SB contributed to the analysis. EMT and SB wrote the manuscript. All authors read and approved the final manuscript.

## Pre-publication history

The pre-publication history for this paper can be accessed here:



## Supplementary Material

Additional file 1 **Scatter-plots of control hybridizations using the MHC tiling array**. a). Comparison of biological replicates; b). Comparison of biological replicates after LM-PCR; c). Comparison of profiles with and without LM-PCR; d). Comparison of dye swaps after LM-PCR. Sperm DNA was used in all comparisons. Correlation coefficients (R^2^) are given for each comparison. Click here for file

Additional file 2 **tDMRs within the MHC region**. A total of 90 tDMRs were identified. Six pair-wise comparisons were performed and, in total, 90 tDMRs were identified using t-statistics (see Methods). tDMRs of each comparison and their co-ordinates on chromosome 6 are provided. M values which are equivalent to the log_2 _ratio of the two corresponding methylation profiles in each comparison are shown. A threshold of p-value <0.001 was used. P-values of the 90 tDMRs are provided. Click here for file
