# Supplementary figures and images for: Generation of a genomic tiling array of the human Major Histocompatibility Complex (MHC) and its application for DNA methylation analysis
Source: BMC Med Genomics. 2008 May 30;1:19. doi: 10.1186/1755-8794-1-19 (PMC2430202; doi:10.1186/1755-8794-1-19)

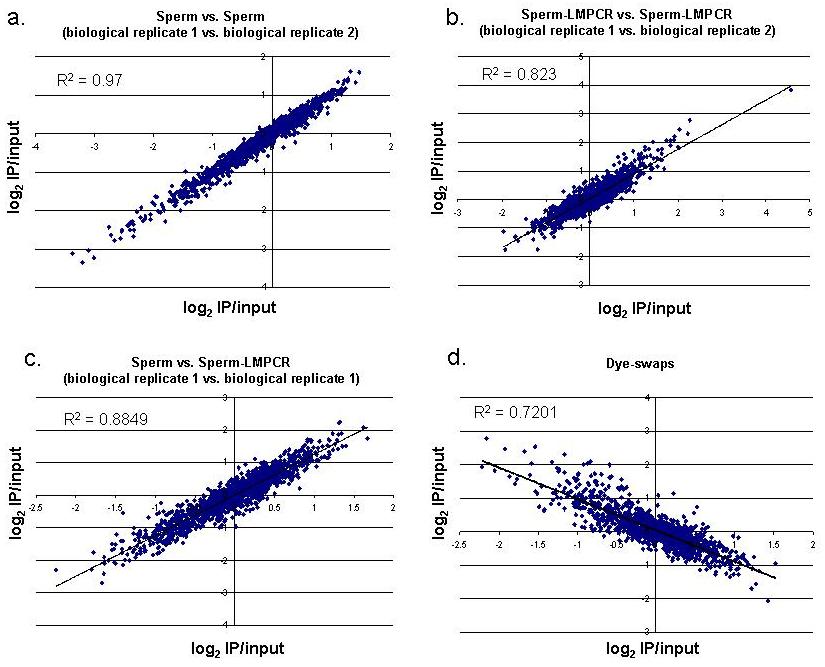

Supplement: Additional file 1 — Scatter-plots of control hybridizations using the MHC tiling array. a). Comparison of biological replicates; b). Comparison of biological replicates after LM-PCR; c). Comparison of profiles with and without LM-PCR; d). Comparison of dye swaps after LM-PCR. Sperm DNA was used in all comparisons. Correlation coefficients (R2) are given for each comparison. [file 1755-8794-1-19-S1.jpeg]
